# Regulations of malaria in children with human immunodeficiency virus infection: A review

**DOI:** 10.1097/MD.0000000000036166

**Published:** 2023-11-17

**Authors:** Abdulrahman Abdulbasit Opeyemi, Emmanuel Ifeanyi Obeagu

**Affiliations:** a Department of Medical Laboratory Science, Achievers University, Owo, Nigeria; b Department of Medical Laboratory Science, Kampala International University, Kampala, Uganda.

**Keywords:** children, human immunodeficiency virus infection, malaria, regulation

## Abstract

This comprehensive review explores the intricate relationship between 2 major global health challenges, malaria and HIV, with a specific focus on their impact on children. These diseases, both endemic in sub-Saharan Africa, create a dual burden that significantly elevates the risk of morbidity and mortality, particularly in children with compromised immune systems due to HIV. The review delves into the complex mechanisms by which these infections interact, from heightened clinical malaria frequencies in HIV-infected individuals to the potential impact of antiretroviral therapy on malaria treatment. Different research engines were utilized in writing this paper such as Web of Science, Google Scholar, Pubmed Central, ResearchGate, and Academia Edu. To address this critical health concern, the study identifies and discusses various regulatory and treatment strategies. It emphasizes the importance of daily cotrimoxazole prophylaxis and insecticide-treated nets in preventing malaria in children with HIV. The potential of antiretroviral protease inhibitors and mRNA-based vaccines as innovative solutions is highlighted. Additionally, the study underscores the significance of climate data and artificial intelligence in improving diagnostics and drug development. Furthermore, the review introduces the concept of genetically modified mosquitoes as a novel approach to vector control, offering a promising avenue to protect HIV-positive individuals from mosquito-borne diseases like malaria. Through a comprehensive analysis of these strategies, the study aims to provide a foundation for policymakers, healthcare professionals, and researchers to develop effective regulations and interventions that reduce the dual burden of malaria and HIV in children, improving public health outcomes in endemic regions.

## 1. Introduction

Among the 2 most significant global health issues facing developing nations are malaria and HIV. Each year, they are the cause of almost 4 million fatalities. Plasmodium is the genus of protozoan parasites that cause malaria, commonly known as the “king of diseases.”^[1]^ Malaria causes more than a million deaths each year, with sub-Saharan Africa accounting for 90% of these deaths. Malaria is the primary cause of mortality for children in this region. An estimated 300 to 500 million Africans contract malaria annually.^[2]^ Because poverty is a major barrier to accessing effective healthcare and is associated with unhygienic living conditions and mosquito breeding grounds, malaria has remained a persistent problem in sub-Saharan Africa.^[2]^

The weakening of the cellular immune system characterizes the human immunodeficiency virus (HIV), an emerging infectious disease agent.^[3]^ With over 36 million HIV-positive individuals and over 1 million HIV-related fatalities in 2017,^[4]^ HIV is a well-known worldwide health burden. Among the most devastating epidemics the world has ever seen is HIV/AIDS. Up to 2.5 million of the estimated 33.2 million HIV-positive individuals living in the world in 2007 were minors, under the age of fifteen. In addition, 420,000 children under the age of 15 had their first HIV infection in 2007. Sub-Saharan Africa is home to over 90% of all HIV-positive children.^[1]^

Due to the different ways that malaria and HIV interact, an individual’s prognosis worsens if they have both infections at the same time. According to Akinyede et al,^[2]^ HIV infection raises the frequency and intensity of clinical malaria. A decrease in T-cell immunity, which is essential for responses against malaria, is the cause of this. Co-infection with HIV and malaria has an impact on a person’s health and nutritional status, potentially increasing their risk of morbidity and mortality. Sanyaolu et al^[5]^ reported that research conducted in Nigeria and Ethiopia revealed that individuals who had both HIV and malaria were more anemic than those who did not have similar infections. Co-infections with HIV and malaria have been linked to decreased immunity and a lower CD4 count.^[6]^ Additionally, adherence to antiretroviral therapy (ART) treatment is associated with a higher risk of death in patients with co-infections with malaria.^[7]^ Adverse birth outcomes, such as low infant birth weights, preterm deliveries, and abortions, are also linked to co-infection.^[7]^ HIV infection causes severe damage and modification in both cellular and humoral immunity as well as resistance to *Plasmodium falciparum* infection, intensifying the effects of malaria in pregnant women and newborns. On the other hand, acute malaria infection raises the viral load, promotes HIV transmission, and accelerates the course of the illness, all of which have significant consequences for public health.^[8]^

There is a reciprocal and cooperative interaction between these 2 illnesses. Increased parasite loads may contribute to greater rates of malaria transmission, and HIV infection might heighten the risk and severity of malaria infection.^[1]^ HIV-positive people who live in malaria-endemic regions but are thought to be semi-immune to the disease may also develop clinical malaria. Additionally, significant CD4 + cell activation and the upregulation of proinflammatory cytokines are linked to malaria infection. These conditions create an excellent milieu for the virus to disseminate among CD4 + cells, which in turn promotes rapid replication of HIV-1.^[1]^

The relevance of both infections to public health cannot be overstated, particularly in areas like sub-Saharan Africa where both diseases are endemic. For this reason, it is crucial to comprehend the regulatory framework that must be in place in order to manage this dual burden when creating focused interventions and policies. Additionally, it is critical that this review address effective regulations and management strategies that are essential to reducing the risk of severe complications, including an increased risk of mortality and morbidity in this vulnerable population, given the compromised immune nature of children with HIV, which renders them susceptible to severe malaria infections. Similarly, the review process will educate policymakers and medical professionals on effective regulatory knowledge regarding possible drug interactions, treatment plans, and methods to maximize the management of both infections while reducing side effects and guaranteeing the effectiveness of antiretroviral therapy, which will ultimately address this health burden. The overall importance of the review is based on its ability to address co-infection of both malaria and HIV in children by providing detailed insights into effective treatment and regulations to reduce mortality and morbidity among the vulnerable young population in area where malaria is mostly endemic. In essence, the review aims to investigate and understand the interaction between malaria and HIV in children, emphasizing the need for tailored interventions, evaluate existing and novel treatment options, including mRNA-based vaccines and AI-driven drug discovery, to combat co-infections effectively and propose regulatory and policy measures to enhance the management of both diseases, considering climate factors and genetic interventions for vector control.

### 1.1. Treating and managing malaria in HIV infected children

HIV and malaria infections, especially those caused by plasmodium parasites, are pathogens that cause a great deal of immune system disruption and activation. HIV and malaria are 2 infections that may have influenced each other’s growth, severity, and rate of disease progression.^[6]^ Given the seriousness of the possible effects of both illnesses on the general population, it will be crucial to comprehend the management and treatment approaches for preventing malaria infection in children with HIV in order to improve overall health outcomes in endemic areas.

The World Health Organisation (WHO) recommends daily cotrimoxazole chemotherapy as a key tactic to avoid opportunistic infections among people living with HIV in Sub-Saharan Africa.^[9]^ In areas where malaria is widely prevalent, the WHO advises continuing cotrimoxazole prophylaxis irrespective of CD4 cell count or WHO clinical stages.^[10]^ Numerous studies have confirmed the assertion that those who did not receive CTX prophylaxis had higher rates of malaria infection than those who had daily cotrimoxazole prophylaxis.^[11,12]^ One possible explanation is that CXT can help infants and adults receiving antiretroviral medication restore their immune systems by treating and avoiding serious bacterial infections and malaria.^[13]^ The efficacy and long-term use of this medication may be restricted due to a potential downside of anti-folate resistance, despite substantial study evidence supporting its powerful antimalarial prophylactic qualities.^[14]^ However, a study by Bigira et al^[15]^ revealed that monthly dihydroartemisinin-piperaquine (DP) was the most effective and safe treatment for preventing malaria in children living in a region of high transmission intensity, but adherence may be an issue. Daily trimethoprim-sulfamethoxazole (TS) and monthly sulfadoxine-pyrimethamine (SP) treatments might not be suitable in regions where anti-folate resistance is common and transmission intensity is high.

Antiretroviral protease inhibitors exhibit efficacy against *P falciparum* in vitro, which is the causative agent of the majority of malaria cases in Africa. This activity is likely due to the inhibition of plasmodial aspartic proteases, which share biochemical similarities with HIV-1 protease.^[16]^ The most effective of these inhibitors is lopinavir, which exhibits activity at much lower levels than those attained with conventional dosages of lopinavir–ritonavir co-formulated.^[17]^ Lopinavir–ritonavir is becoming more widely available in Africa for the treatment of HIV infections, which suggests that it could be a useful weapon in the fight against malaria. However, because ritonavir affects many medications’ metabolisms, it may interact with antimalarial medications, such as popular artemisinin-based combination regimens, thereby compromising the medications’ safety and efficacy.^[18]^

Using insecticide-treated nets (ITNs) is thought to be another best way to prevent malaria among children infected with HIV. Even in locations with high HIV prevalence, randomized controlled studies showed that community-level usage of ITNs was linked to a 50% decrease in malaria incidence and a 16% decrease in all-cause mortality in children under the age of 5.^[19]^ According to Yibeltal et al,^[7]^ households that regularly used insecticide residual spray and used an ITN at bedtime were shown to have a lower risk of contracting malaria. According to a post-intervention assessment, the long-lasting insecticide-treated net (LLITN) decreased the incidence of malaria among pregnant and lactating women in Nigeria from 35% to 6.0% 6 months after the intervention.^[11]^ Likewise, research conducted in Ethiopia’s Northern and Oromia regions revealed that PLWHA who used ITN had a lower chance of contracting malaria. One possible explanation is that bed nets sprayed with insecticides kill mosquitoes and serve as physical barriers in addition to having a repellent impact on their numbers.^[20]^

### 1.2. Regulations of malaria in children with HIV

Even in PLWHA, malaria can be efficiently treated.^[1]^ The necessity of expanding this intervention is underscored by the fact that, despite resistance to some anti-malarial medications having occurred in some parts of the world, resistance to artemisinin-based combination therapy (ACT) has not developed.^[9]^ Chloroquine and primaquine should be used to treat *P. vivax* infections, unless the latter is resistant to the former.^[21]^ An ACT should be used to treat any simple *P falciparum* infections. Additionally, prompt ACT treatment can lessen transmission.^[21]^ HIV and malaria programmes can complement one another to improve laboratory services for diagnosis, bolster health systems, enhance supply chains and distribution networks, and reach more people in the community with health services. In order to do so, the following potential regulations must be taken into account^[22]^:

1. In order to encourage women and children under 5 to go to bed under LLINs every night, public education programmes and Information, Education, and Communication (IEC) materials should be integrated into HIV/AIDS and Malaria programmes.
2. In order to maximize resource utilization and impact on co-infected individuals, efforts to increase net usage for malaria and to distribute LLINs should be integrated into programmes for voluntary counseling and examination (VCE), antenatal care, and the avoidance of transmission from mother to child (PMTCT) in all malaria-endemic countries.
3. It is imperative to guarantee the prompt evaluation and therapy of febrile patients with antiretrovirals (ACTs) in areas where HIV/AIDS and malaria are coexisting.
4. To ensure a strong, community-focused response to malaria, HIV/AIDS programmes and initiatives at the level of the community should be incorporated, and information should be shared in a way that promotes and improves the work of the National Malaria Control Plans.
5. Governments must guarantee higher levels of financing in order to meet international commitments to obtaining widespread access to treatment for PLWHA and the provision of LLINs for malaria treatment as well as prevention.
6. National governments should work to improve coordination among partners and expand access by involving more non-governmental, civil society, and religious organizations in cooperative HIV/AIDS and malaria programmes and raising the possibility that these partners will become sub-recipients of Global Fund grants.

### 1.3. Identifying novel treatments and regulations for the management of malaria in HIV

Based on the deadliness of both infections, it is important for this review to identify novel treatment targets that can aid in the prevention and treatment of people living with HIV. It is with such effort that it is important for researchers, health agencies, and government bodies to look into mRNA-based vaccines against malaria parasites. This is due to the fact that vaccination is unquestionably one of the biggest medical advancements, saving millions of lives from infectious diseases every year, and mRNA-based vaccines have demonstrated their efficacy during the COVID-19 pandemic.^[23]^ The mRNA-LNP vaccines targeting *P falciparum* glutamic acid-rich protein (PfGARP), mosquito saliva protein AgTRIO, and Cell-Traversal Protein for Ookinetes and Sporozoites (CelTOS), PfCSP, and Pfs25 protein constituents that enhance ookinetes and oocyst formation have proven to demonstrate effectiveness against malaria parasites through various mechanisms, such as inhibition of the life cycle of the parasite in both mosquito and human host. Thus preventing replication and transmission of the infection.^[23,24]^ As such, the government and research institutes must be willing to provide funding in support of research around this novel target that will enhance the development of a vaccine that will confer immunity against malaria infection and encourage those living with HIV to live without fear of suffering from co-infection in malaria-endemic regions.

In order to ensure laboratory policies that improve both RDT and microscopic diagnosis of malaria parasite infection among people living with HIV, various hospital managements across the malaria-endemic region must implement effective policies. These will ensure an accurate diagnosis of malaria while ensuring the difference between malaria-related fever and non-malaria-related fever in such regions, as people living with HIV may present with non-malaria-related fever based on their immune status. More so, the 2 diagnostic methods will complement each other’s strengths and limitations and enhance a vital malaria control program among people living with HIV.^[25]^

Another important aspect is climate action. Due to the climate-sensitive nature of malaria infection that supports its vector host, there is a need for climate-driven models and regulations targeted at weather forecasts around temperature, rainfall, and humidity, which may directly or indirectly affect malaria vectors in endemic regions.^[26]^ As such, it is important for climate data to be generated and communicated at intervals to the populace to provide broad indications of transmission potential variability and provide information to strengthen malaria control interventions among people living with HIV. However, care must be taken with the use of climate-driven data to avert climate shock.^[27]^

Artificial intelligence-based computational modeling, simulation, and prediction techniques are growing in strength and accuracy. They are also very accessible, which is especially helpful for researchers from developing nations who want to help pinpoint malaria hotspots and develop HIV prevention strategies. Researchers in these areas have found antimalarial drugs that work in different ways. They did this using a variety of techniques, such as Naïve Bayesian (NB), Support Vector Machine (SVM), Random Forest (RF), and drug targets (DT). These techniques improve the quality, efficiency, and effectiveness of newly developed drugs.^[28]^ Various data sets, particularly against the active compound of plasmodium and HIV infection, have been individually generated.^[29]^ But then, there is a need for a comprehensive target against co-infection in people living with HIV through these methods.

There is no doubt that new, complementary approaches are needed to close the gaps in current vector control interventions, such as effective control of outdoor biting, and to provide alternatives to manage the increasing threat of insecticide resistance. However, a cost-effective intervention through genetically modified mosquitoes may be effective in complementing existing interventions.^[30]^ These genetically derived mosquitoes inherit genes from generation to generation, allowing for the introduction of new traits through interbreeding. These systems aim to reduce vector mosquito populations by inhibiting reproduction or survival or by making them less competent to transmit pathogens, known as gene drive systems.^[31]^ In an effort to protect HIV-positive individuals from mosquito-borne illnesses like malaria, genetically modified insects are being produced as potential new instruments. None of these mosquitoes have been tested in the field as of yet. Considering the potential regulatory requirements for the eventual deployment of these technologies in national or regional public health programs at this early stage is advantageous for designers, advocates, and interested parties. This is because some of the practical implications of these requirements may require significant planning, time, and integration to address.^[32]^

## 2. Conclusion

This review underscores the urgent need for a multi-faceted approach to address the co-occurrence of malaria and HIV, particularly in children residing in malaria-endemic regions of sub-Saharan Africa. The intertwined nature of these diseases, their impact on the immune system, and their complex interactions demand comprehensive regulatory and treatment strategies to mitigate their combined burden effectively.

The evidence supports the World Health Organization’s recommendation of daily cotrimoxazole prophylaxis as a crucial element in preventing malaria in children with HIV. This intervention not only reduces malaria incidence but also aids in restoring immune function, especially when combined with antiretroviral therapy. However, the potential limitation of anti-folate resistance underscores the need for continuous monitoring and research into alternative prophylactic treatments such as monthly dihydroartemisinin-piperaquine.

Furthermore, antiretroviral protease inhibitors, particularly lopinavir, exhibit promise in managing both HIV and malaria. Their ability to inhibit *P falciparum* and reduce malaria transmission makes them valuable tools in this dual battle. However, careful consideration of potential drug interactions is necessary, emphasizing the importance of an interdisciplinary approach to optimize treatment regimens.

Insecticide-treated nets (ITNs) are proven interventions that can significantly reduce malaria incidence, even in regions with a high HIV prevalence. Their combination with insecticide residual sprays creates a potent defense against mosquito-borne diseases. Such integrated strategies must be embedded within broader healthcare programs to ensure a holistic approach to the management of both infections.

Effective regulations and policies are critical to achieving these goals. Public education campaigns, integrated into HIV/AIDS and malaria programs, can encourage the use of ITNs, ensuring higher adherence rates. Collaboration among governments, NGOs, civil society, and religious organizations is vital for expanding access to treatment and prevention services. Financial commitments must be met to facilitate widespread access to antiretroviral therapy and malaria prevention measures, including the distribution of long-lasting insecticide-treated nets.

Climate data, as a tool to predict malaria transmission dynamics, is invaluable for improving malaria control efforts. Coupled with artificial intelligence-driven drug discovery, these tools enhance our ability to develop targeted interventions and pinpoint malaria hotspots. Moreover, novel approaches, such as genetically modified mosquitoes and mRNA-based vaccines, hold promise for the future, but thorough research and careful regulation are essential before their deployment.

In all, tackling the dual burden of malaria and HIV in children is a complex endeavor that necessitates a comprehensive, multifaceted strategy. Effective regulations, innovative treatment options, and enhanced preventive measures are crucial for reducing morbidity and mortality in this vulnerable population, thereby mitigating the public health challenges posed by these 2 interrelated diseases.

## Author contributions

**Conceptualization:** Emmanuel Ifeanyi Obeagu.

**Methodology:** Abdulrahman Abdulbasit Opeyemi, Emmanuel Ifeanyi Obeagu.

**Supervision:** Emmanuel Ifeanyi Obeagu.

**Validation:** Emmanuel Ifeanyi Obeagu.

**Visualization:** Emmanuel Ifeanyi Obeagu.

**Writing – original draft:** Abdulrahman Abdulbasit Opeyemi, Emmanuel Ifeanyi Obeagu.

**Writing – review & editing:** Abdulrahman Abdulbasit Opeyemi, Emmanuel Ifeanyi Obeagu.

## References

[1] Alemu A Shiferaw Y Addis Z. Effect of malaria on HIV/AIDS transmission and progression. Parasites Vectors. 2013;6:18. 23327493 10.1186/1756-3305-6-18 PMC3564906

[2] Akinyede AA Akintonwa A Okany C. Knowledge, treatment seeking and preventive practices in respect of malaria among patients with HIV at the Lagos University Teaching Hospital. Tanzania J Health Res. 2011;13. 10.4314/thrb.v13i4.63347 26592054

[3] Mirzohreh ST Safarpour H Pagheh AS. Malaria prevalence in HIV-positive children, pregnant women, and adults: a systematic review and meta-analysis. Parasites Vectors. 2022;15:324. 36104731 10.1186/s13071-022-05432-2 PMC9472338

[4] Frank TD Carter A Jahagirdar D. Global, regional, and national incidence, prevalence, and mortality of HIV, 1980–2017, and forecasts to 2030, for 195 countries and territories: a systematic analysis for the Global Burden of Diseases, Injuries, and Risk Factors Study 2017. Lancet HIV. 2019;6:e831–59. 31439534 10.1016/S2352-3018(19)30196-1 PMC6934077

[5] Sanyaolu A Fagbenro-Beyioku A Oyibo W. Malaria and HIV co-infection and their effect on haemoglobin levels from three healthcare institutions in Lagos, southwest Nigeria. Afr Health Sci. 2013;13. 10.4314/ahs.v13i2.14 PMC3824477 24235927

[6] Jegede FE Oyeyi TI Abdulrahman SA. Effect of HIV and malaria parasites co-infection on immune-hematological profiles among patients attending anti-retroviral treatment (ART) clinic in Infectious Disease Hospital Kano, Nigeria. PLoS One. 2017;12:e0174233. 28346490 10.1371/journal.pone.0174233 PMC5367709

[7] Yibeltal T Dereje BA Amsalu BM. Determinants of HIV-malaria co-infection among people living with HIV on antiretroviral therapy in Northeast Ethiopia: unmatched case control study. Trop Med Health. 2020;48:94. 33292761 10.1186/s41182-020-00286-9 PMC7702680

[8] Dibua UME Lorina BE Joseph AU. HIV and malaria co-infection: Their combined effects on pregnancy outcomes in Anambra State, Southeast Nigeria. Int J Med Medical Sci. 2013;5:438–49.

[9] World Health Organisation. Consolidated Guidelines on the Use of Antiretroviral Drugs for Treating and Preventing HIV Infection: Recommendations for a Public Health Approach. WHO Guidelines. 2013; 272. 24716260

[10] WHO. Consolidated Guidelines on the Use of Antiretroviral Drugs for Treating and Preventing HIV Infection. 2nd ed. 2016. 27466667

[11] Beyene HB Tadesse M Disassa H. Concurrent Plasmodium infection, anemia and their correlates among newly diagnosed people living with HIV/AIDS in Northern Ethiopia. Acta Trop. 2017;169:8–13. 28119046 10.1016/j.actatropica.2017.01.007

[12] Alemayehu G Melaku Z Abreha T. Burden of malaria among adult patients attending general medical outpatient department and HIV care and treatment clinics in Oromia, Ethiopia: a comparative cross-sectional study. Malar J. 2015;14:501. 26671012 10.1186/s12936-015-1029-0 PMC4681039

[13] World Health Organization. March 2014 Supplement to the 2013 Consolidated Guidelines on the Use of Antiretroviral Drugs for Treating and Preventing HIV Infection: Recommendations for a Public Health Approach. World Health Organization. 2014. Available at: https://iris.who.int/handle/10665/104264. 24716260

[14] Manyando C Njunju EM D’Alessandro U. Safety and efficacy of co-trimoxazole for treatment and prevention of *Plasmodium falciparum* malaria: a systematic review. PLoS One. 2013;8:e56916. 23451110 10.1371/journal.pone.0056916 PMC3579948

[15] Bigira V Kapisi J Clark TD. Protective efficacy and safety of three antimalarial regimens for the prevention of malaria in young Ugandan children: a randomized controlled trial. PLoS Med. 2014;11:e1001689. 25093754 10.1371/journal.pmed.1001689 PMC4122345

[16] Nsanzabana C Rosenthal PJ. In vitro activity of antiretroviral drugs against *Plasmodium falciparum*. Antimicrob Agents Chemother. 2011;55:5073–507. 21876053 10.1128/AAC.05130-11 PMC3194998

[17] Achan J Kakuru A Ikilezi G. Antiretroviral agents and prevention of malaria in HIV-infected Ugandan children. N Engl J Med. 2012;367:2110–8. 23190222 10.1056/NEJMoa1200501 PMC3664297

[18] German P Parikh S Lawrence J. Lopinavir/ritonavir affects pharmacokinetic exposure of artemether/lumefantrine in HIV-uninfected healthy volunteers. JAIDS J Acquir Immune Defic Syndr. 2009;51:424–9. 19506482 10.1097/QAI.0b013e3181acb4ff

[19] Kamya MR Byakika-Kibwika P Gasasira AF. The effect of HIV on malaria in the context of the current standard of care for HIV-infected populations in Africa. Future Virol. 2012;7:699–708. 23293660 10.2217/FVL.12.59 PMC3535690

[20] Walson JL Sangaré LR Singa BO. Evaluation of impact of long-lasting insecticide-treated bed nets and point-of-use water filters on HIV-1 disease progression in Kenya. AIDS. 2013;27:1493–501. 23324658 10.1097/QAD.0b013e32835ecba9

[21] CDC. *CDC – Malaria – Diagnosis & Treatment (United States) – Treatment (US) - Guidelines for Clinicians*. Centers Dis Control Prev. 2020. Available at: https://www.cdc.gov/malaria/diagnosis_treatment/clinicians1.html.

[22] World V. *The Link between Malaria and HIV and AIDS Background*. 2023; https://www.wvi.org/sites/default/files/HIV_and_Malaria_flyer_English.pdf.

[23] Matarazzo L Bettencourt PJG. mRNA vaccines: a new opportunity for malaria, tuberculosis and HIV. Front Immunol. 2023;14:1–5. 10.3389/fimmu.2023.1172691 PMC10166207 37168860

[24] Tsoumani ME Voyiatzaki C Efstathiou A. Malaria vaccines: from the past towards the mRNA vaccine era. Vaccines. 2023;11:1452. 37766129 10.3390/vaccines11091452 PMC10536368

[25] Andreoli A Giorgetti PF Pietra V. Evaluation of a PfHRP-2 based rapid diagnostic test versus microscopy method among HIV-positive and unknown serology patients in Ouagadougou, Burkina Faso. Am J Trop Med Hyg. 2015;92:834–7. 25667051 10.4269/ajtmh.13-0757 PMC4385783

[26] Rocklöv J Dubrow R. Climate change: an enduring challenge for vector-borne disease prevention and control. Nat Immunol. 2020;21:479–83. 32313242 10.1038/s41590-020-0648-y PMC7223823

[27] Nissan H Ukawuba I Thomson M. Climate-proofing a malaria eradication strategy. Malar J. 2021;20:215. 33865383 10.1186/s12936-021-03718-x PMC8053291

[28] Patel L Shukla T Huang X. Machine learning methods in drug discovery. Molecules. 2022;25:5277. 10.3390/molecules25225277 PMC7696134 33198233

[29] Winkler DA. Use of artificial intelligence and machine learning for discovery of drugs for neglected tropical diseases. Front Chem. 2021;9:614073. 33791277 10.3389/fchem.2021.614073 PMC8005575

[30] World Health Organization. WHO issues new guidance for research on genetically modified mosquitoes to fight malaria and other vector-borne diseases, 2021. Www.who.int. Available at: https://www.who.int/news/item/19-05-2021-who-issues-new-guidance-for-research-on-genetically-modified-mosquitoes-to-fight-malaria-and-other-vector-borne-diseases.

[31] Alphey LS Crisanti A Randazzo F. Opinion: standardizing the definition of gene drive. Proc Natl Acad Sci USA. 2020;202020417. 10.1073/pnas.2020417117 PMC7733814 33208534

[32] James SL Dass B Quemada H. Regulatory and policy considerations for the implementation of gene drive-modified mosquitoes to prevent malaria transmission. Transgenic Res. 2023;32:17–32. 36920721 10.1007/s11248-023-00335-z PMC10102045

